# Kidney function, proteinuria and breast arterial calcification in women without clinical cardiovascular disease: The MINERVA study

**DOI:** 10.1371/journal.pone.0210973

**Published:** 2019-01-17

**Authors:** Rishi V. Parikh, Carlos Iribarren, Catherine Lee, Tory Levine-Hall, Thida C. Tan, Gabriela Sanchez, Huanjun Ding, Fatemeh Azamian Bidgoli, Sabee Molloi, Alan S. Go

**Affiliations:** 1 Division of Research, Kaiser Permanente Northern California, Oakland, CA, United States of America; 2 Department of Radiological Sciences, University of California Irvine School of Medicine, Irvine, CA, United States of America; 3 Departments of Epidemiology, Biostatistics and Medicine, University of California at San Francisco, San Francisco, CA, United States of America; International University of Health and Welfare, School of Medicine, JAPAN

## Abstract

**Background:**

Breast arterial calcification (BAC) may be a predictor of cardiovascular events and is highly prevalent in persons with end-stage kidney disease. However, few studies to date have examined the association between mild-to-moderate kidney function and proteinuria with BAC.

**Methods:**

We prospectively enrolled women with no prior cardiovascular disease aged 60 to 79 years undergoing mammography screening at Kaiser Permanente Northern California between 10/24/2012 and 2/13/2015. Urine albumin-to-creatinine ratio (uACR), along with specific laboratory, demographic, and medical data, were measured at the baseline visit. Baseline estimated glomerular filtration rate (eGFR), medication history, and other comorbidities were identified from self-report and/or electronic medical records. BAC presence and gradation (mass) was measured by digital quantification of full-field mammograms.

**Results:**

Among 3,507 participants, 24.5% were aged ≥70 years, 63.5% were white, 7.5% had eGFR <60 ml/min/1.73m^2^, with 85.7% having uACR ≥30 mg/g and 3.3% having uACR ≥300 mg/g. The prevalence of any measured BAC (>0 mg) was 27.9%. Neither uACR ≥30 mg/g nor uACR ≥300 were significantly associated with BAC in crude or multivariable analyses. Reduced eGFR was associated with BAC in univariate analyses (odds ratio 1.53, 95% CI: 1.18–2.00), but the association was no longer significant after adjustment for potential confounders. Results were similar in various sensitivity analyses that used different BAC thresholds or analytic approaches.

**Conclusions:**

Among women without cardiovascular disease undergoing mammography screening, reduced eGFR and albuminuria were not significantly associated with BAC.

## Introduction

Arterial calcification is a prominent predictor of cardiovascular morbidity and mortality, and is common among patients with advanced chronic kidney disease (CKD) and end-stage renal disease (ESRD).[1–5] Several studies have estimated the prevalence of arterial calcification in selected vascular beds to be approximately 60–70% in the CKD population, which independently increases the risk of cardiovascular disease (CVD) events by approximately 40%.[6, 7] Calcification of the intima of coronary arteries and aorta, specifically, has been the focus of previous work and has been consistently associated with an increase in atherosclerotic CVD events.[8, 9] However, both intimal (atherosclerotic) and medial (arteriosclerotic or Möneckberg’s sclerosis) calcification are present in the CKD population. It remains unclear whether intimal vs. medial calcification have similar strength of association with CVD risk, especially given differences in pathology between these forms of arterial calcification.[10–12]

Breast arterial calcification (BAC) can be observed on routine mammography and is considered to be primarily medial calcification.[13] Prior studies have found that the prevalence of BAC among women in screening programs is between 10 to 12%, and has been reported to be associated with a 30–40% relative increase in CVD events and death in selected populations.[14] BAC has been consistently found to be strongly associated with increasing age, and is also more prevalent among patients with diabetes mellitus. Reproductive factors, including number of children, breastfeeding, the use of hormone replacement therapy, and menopause, have all been associated with an increase in BAC prevalence. However, associations between BAC and several known cardiovascular risk factors such as hypertension, obesity, dyslipidemia, and smoking remain unclear or negative, possibly suggesting an etiology of CVD separate from classical atherosclerosis.[14–18]

Similar to coronary artery calcification, BAC has been found to be more prevalent among patients with advanced CKD or ESRD treated with chronic dialysis, although limited evidence exists for an independent or graded relationship between less severe kidney dysfunction and BAC.[17, 19–21] The prevalence of BAC in the advanced CKD population is between 40–60%, much higher than the general population.[14] However, it remains unclear if kidney function or damage is linked with BAC independent of other identified risk factors in women (e.g., age, reproductive history, other clinical CVD risk factors). Furthermore, previous studies of BAC have used less precise measurement methods based on the presence or absence of BAC by visual, non-quantitative inspection of mammograms or computed tomography scans.[13, 14, 17, 19] Given that mammograms are conducted in a large fraction of women in the U.S. to screen for breast cancer, an approach to evaluating BAC quantitatively and determining its prognostic significance is attractive as a potential way to enhance CVD risk stratification. In addition, given that different associations exist between kidney dysfunction and various vascular beds, understanding whether renal function and damage are associated with BAC could have important implications.

Toward that end, we explored the independent association between measures of kidney function and damage with a novel, continuous measure of BAC mass in women with no prior cardiovascular disease undergoing screening mammography.

## Materials and methods

### Source population

The source population was based in Kaiser Permanente Northern California (KPNC), a large, integrated healthcare delivery system that currently provides comprehensive care for >4.2 million members. The KPNC membership is highly representative of the local surrounding and statewide population in terms of age, gender and race/ethnicity.[22] Nearly all aspects of care are captured through KPNC’s Epic-based electronic medical record system that is integrated across all practice settings.

This study was approved by the institutional review boards of Kaiser Permanente Northern California and University of California, Irvine, and all participants provided written informed consent.

### Study sample

The MultIethNic study of brEast aRterial calcium gradation and cardioVAscular disease (MINERVA) is a prospective cohort study of women aged 60 to 79 years without known clinical cardiovascular disease who were receiving regular mammography screening.[23] Patients were recruited at one of nine KPNC facilities between October 24, 2012 and February 13, 2015. Patients were excluded if they were age <60 or >79 years at the time of mammographic screening (N = 127,729); had a documented history of myocardial infarction, coronary revascularization, ischemic stroke, heart failure, or peripheral artery disease (N = 22,343); had a history of breast cancer, mastectomy or receipt of breast implants (N = 4,216); diagnosed dementia (N = 374); ESRD defined as receipt of chronic dialysis or renal transplant (N = 42); or had no assigned primary care provider (N = 105). Enrolled participants underwent a baseline study visit where we obtained demographic, behavioral and clinical data using a self-administered questionnaire, and performed selected clinical procedures and laboratory tests. For the present analysis, we further excluded patients who had no baseline visit and no urine albumin-to-creatinine ratio (uACR) or estimated glomerular filtration (eGFR) test results (N = 42,605).

### Kidney function and proteinuria

Our primary predictors of interest were eGFR calculated using the CKD-EPI equation[24] based on the most recent outpatient, non-emergency-department serum creatinine measurement on or within one year before the study visit,[25] as well as proteinuria reflected using a spot uACR measured at the baseline study visit in a CLIA-approved regional health plan laboratory.

### Breast arterial calcification

Our main outcome was BAC measured by analysis of full-field digital mammograms (Senographe 2000D, General Electric Medical Systems, Milwaukee, WI or Selenia Hologic). Standard digital mammograms were acquired by trained mammography technicians and transmitted using a secure FTP connection to a workstation for image analysis at the Department of Radiological Sciences, University of California, Irvine. Using mediolateral oblique and craniocaudal projections, arterial calcium mass was quantified (in mg) in each projection and then summed to obtain an estimate of total BAC burden.[26]

### Covariates

Demographic characteristics, social history and lifestyle factors, and selected comorbidities were obtained through the self-administered questionnaire at the baseline visit. Laboratory results, including serum calcium, high sensitivity C-reactive protein (hs-CRP), glycosylated hemoglobin (HbA1C), hemoglobin, low density lipoprotein (LDL) cholesterol, phosphorous, parathyroid hormone (PTH), and vitamin D, were also collected at the baseline visit. Other relevant cardiovascular and medical comorbidities were defined by diagnostic or procedure *International Classification of Diseases*, *Ninth Edition*, *Clinical Modification* (ICD-9-CM) codes and supplemented with laboratory test results (e.g., blood glucose), outpatient vital signs, and/or prescribed medications using electronic health record-based data that were linked at the individual-patient level into the Kaiser Permanente Northern California Virtual Data Warehouse (KPNC VDW) as previously described and validated.[27–39] Diabetes mellitus was identified if patients met one or more of the following conditions: one or more primary inpatient discharge diagnoses of ICD-9 code 250.X, two or more outpatient or emergency discharge diagnoses of ICD-9 code 250.X, presence of an abnormal outpatient blood glucose laboratory value, or one or more prescriptions of either an insulin, oral hypoglycemic agent, antihyperglycemic agent, or other antidiabetic agent. Furthermore, supposed cases of gestational diabetes or suspected non-diabetes-related metformin or thiazolidinedione use were excluded. Hypertension was identified if patients had at least two outpatient diagnoses of hypertension, or one outpatient diagnosis when paired with an antihypertensive medication.

### Statistical approach

All analyses were conducted using SAS, version 9.3 (Cary, N.C.). Baseline characteristics were compared across presence (BAC >0 mg) or absence (BAC = 0 mg) of BAC using ANOVA for continuous variables and χ^2^ tests for categorical variables.

We next conducted nested logistic regression models for two thresholds of BAC (>0 and BAC ≥10 mg), as well as ordinal logistic regression using tertiles of the BAC distribution among the subset of patients with BAC >0 mg. We categorized eGFR as a binary variable at <60 vs. ≥60 mL/min/1.73 m^2^, and uACR was classified as a binary variable using two primary cutoffs: ≥30 mg/g and ≥300 mg/g, which is consistent with clinical microalbuminuria and macroalbuminuria, respectively. We also examined the association between kidney dysfunction and BAC >0 mg stratified by race/ethnicity.

The series of nested models progressively adjusted for additional categories of potential confounders and explanatory variables. Sociodemographic variables included age, race, educational attainment, household income, marital status, and whether the patient was born in the US. Lifestyle factors included use of alcohol in the past year, smoking status, and hours of physical activity per week. Reproductive history included age at menarche, age periods stopped, polycystic ovary syndrome, receipt of menopausal hormone therapy, history of breastfeeding, and number of children. Prior history of cardiovascular risk factors and diseases, ascertained from records in the KPNC VDW, included atrial fibrillation or flutter, mitral and/or aortic valvular disease, diabetes mellitus, hypertension, and dyslipidemia, as well as ankle-brachial index measured at the baseline visit. Medical history identified from the MINERVA baseline survey included prior migraine, gout, osteoporosis, osteoarthritis, depression, self-reported health status, and parental history of stroke, heart failure, and heart attack. Other medical history identified from the VDW records included hyperthyroidism, hypothyroidism, cirrhosis, chronic lung disease, and hospitalized bleeds. Baseline medication use included prior receipt of angiotensin converting enzyme (ACE) inhibitors, angiotensin II receptor blockers (ARBs), β-blockers, calcium channel blockers, diuretics, aldosterone receptor antagonists, alpha blockers, antiarrhythmic agents, nitrates, other vasodilators, non-aspirin antiplatelet agents, statins, other lipid-lowering agents, anti-diabetic agents, and non-steroidal anti-inflammatory drugs based on pharmacy dispensing within 30 days before or on the baseline visit date. Lastly, we hypothesized that a category of potential biological mediators may help to explain any observed independent relationship between kidney function or damage with BAC; this included systolic blood pressure, body mass index, waist circumference, and calcium, hs-CRP, HBA1c, hemoglobin, LDL cholesterol, phosphorous, parathyroid hormone, and vitamin D levels.

In a sensitivity analysis using linear regression, we modeled log-transformed BAC (specifically, log(BAC + 0.05)) because the distribution of BAC among participants was non-normally distributed. To account for the high prevalence of patients with no detected BAC, we fit loess curves for continuous eGFR, log(uACR) and log(BAC + 0.05) among only those with BAC >0 mg.

Lastly, we conducted another sensitivity analysis using a case-control approach matching CKD patients to non-CKD patients at a 1:1 ratio on age group (60–64, 65–69, 70–74, 75–79) and race category (White, Black, Latina, Asian/Pacific Islander, Other). After matching, odds ratios and corresponding McNemar’s exact 95% confidence limits were calculated.

## Results

### Baseline characteristics

Among 5,145 enrolled participants, we identified a final analytic sample of 3,507 women who met eligibility criteria. Approximately two thirds of the sample was white, with a majority having at least a bachelor’s degree (73.3%), mean household income greater than $60,000/year (66.0%), married (54.7%), born in the US (78.3%), drank alcohol in the past year (79.9%), and non-smokers (61.3%)(**Table 1**). Women with any measured BAC were more likely than women with no BAC to be older; have lower educational attainment and mean household income; to have used alcohol in the past year; had more children; had a prior history of gout, osteoporosis, atrial flutter or fibrillation, hypertension, chronic lung disease, and hospitalized bleeds; have a higher systolic blood pressure, and have a lower baseline eGFR level.

**Table 1:** Baseline characteristics of 3507 women aged 60 to 79 years enrolled in the MINERVA study.

Characteristic	Overall (N = 3507)	Any breast calcification (N = 978)	No breast calcification (N = 2529)	*p*-value
**Demographics and social history**				**<0.001**
Age group, yr				
60–64	1392 (39.7)	282 (28.8)	1110 (43.9)	
65–69	1255 (35.8)	348 (35.6)	907 (35.9)	
70–74	669 (19.1)	245 (25.1)	424 (16.8)	
75–79	191 (5.4)	103 (10.5)	88 (3.5)	
Race				**<0.05**
White	2228 (63.5)	644 (65.8)	1584 (62.6)	
Black	412 (11.7)	105 (10.7)	307 (12.1)	
Latina	324 (9.2)	104 (10.6)	220 (8.7)	
Asian/Pacific Islander	495 (14.1)	113 (11.6)	382 (15.1)	
Other	48 (1.4)	12 (1.2)	36 (1.4)	
Highest educational attainment				**<0.001**
Less than High School	66 (1.9)	27 (2.8)	39 (1.5)	
High School	559 (15.9)	190 (19.4)	369 (14.6)	
Bachelor’s Degree	1529 (43.6)	431 (44.1)	1098 (43.4)	
Graduate School	1077 (30.7)	251 (25.7)	826 (32.7)	
Other	276 (7.9)	79 (8.1)	197 (7.8)	
Household income				**<0.001**
Less than $20,000	150 (4.3)	49 (5.0)	101 (4.0)	
$20,000 - $59,999	861 (24.6)	282 (28.8)	579 (22.9)	
$60,000 - $119,999	1452 (41.4)	389 (39.8)	1063 (42.0)	
$120,000 and greater	864 (24.6)	220 (22.5)	644 (25.5)	
Unknown	180 (5.1)	38 (3.9)	142 (5.6)	
Marital status				0.49
Married	1920 (54.7)	549 (56.1)	1371 (54.2)	
Widowed	328 (9.4)	96 (9.8)	232 (9.2)	
Divorced/Separated	766 (21.8)	210 (21.5)	556 (22.0)	
Never Married	296 (8.4)	79 (8.1)	217 (8.6)	
Living with Someone	155 (4.4)	36 (3.7)	119 (4.7)	
Other	42 (1.2)	8 (0.8)	34 (1.3)	
Born in the USA	2746 (78.3)	760 (77.7)	1986 (78.5)	0.6
Used alcohol in the past year	2628 (74.9)	707 (72.3)	1921 (76.0)	**<0.05**
Smoking status				0.99
Never	2151 (61.3)	597 (61.0)	1554 (61.5)	
Former	1220 (34.8)	344 (35.2)	876 (34.6)	
Current	136 (3.9)	37 (3.8)	99 (3.9)	
Physical activity per week, hrs				
Mean (SD)	6.2 (4.8)	6.0 (4.9)	6.3 (4.8)	0.09
Median (IQR)	5.5 (2.5–9.0)	5.5 (2.5–8.5)	5.5 (2.5–9.0)	0.06
Range	0.0–30.0	0.0–29.0	0.0–30.0	
**Medical history**				
Self-reported health				**<0.05**
Excellent	603 (17.2)	159 (16.3)	444 (17.6)	
Very Good	1614 (46.0)	451 (46.1)	1163 (46.0)	
Good	1080 (30.8)	287 (29.3)	794 (31.4)	
Fair	199 (5.6)	76 (7.8)	122 (4.8)	
Poor	11 (0.3)	5 (0.5)	6 (0.2)	
Age at menarche				0.26
Less than 10 years	79 (2.3)	24 (2.5)	55 (2.2)	
10–11 years	684 (19.5)	206 (21.1)	478 (18.9)	
12–13 years	1878 (53.6)	508 (51.9)	1370 (54.2)	
14–15 years	670 (19.1)	179 (18.3)	491 (19.4)	
16 years or older	194 (5.5)	60 (6.1)	134 (5.3)	
Never had a menstrual period	1 (0.0)	1 (0.1)	0 (0.0)	
Age periods stopped, yr				
Mean (SD)	49.5 (6.3)	49.4 (6.8)	49.5 (6.1)	0.61
Median (IQR)	50.0 (47.0–54.0)	50.0 (46.0–54.0)	50.0 (47.0–54.0)	1.0
Range	25.0–70.0	26.0–70.0	25.0–66.0	
Polycystic ovary syndrome	82 (2.3)	24 (2.5)	58 (2.3)	0.78
Currently on menopausal hormone therapy	420 (12.0)	102 (10.4)	318 (12.6)	0.08
History of breastfeeding	1946 (55.5)	579 (59.2)	1367 (54.1)	**<0.01**
Number of children				**<0.001**
0	956 (27.3)	210 (21.5)	746 (29.5)	
1	601 (17.1)	147 (15.0)	454 (18.0)	
2	1210 (34.5)	332 (34.0)	878 (34.7)	
3	490 (14.0)	164 (16.8)	326 (12.9)	
4	173 (4.9)	76 (7.8)	97 (3.8)	
≥5	77 (2.2)	49 (5.0)	28 (1.1)	
Parental history of stroke	1179 (33.6)	314 (32.1)	865 (34.2)	**0.24**
Parental history of heart failure	1156 (33.0)	322 (32.9)	834 (33.0)	0.98
Parental history of heart attack	1300 (37.1)	365 (37.3)	935 (37.0)	0.85
Migraine	686 (19.6)	194 (19.8)	492 (19.5)	0.8
Gout	104 (3.0)	33 (3.4)	71 (2.8)	**0.37**
Osteoporosis	659 (18.8)	207 (21.2)	452 (17.9)	**<0.05**
Osteoarthritis	869 (24.8)	259 (26.5)	610 (24.1)	0.15
Depression	830 (23.7)	223 (22.8)	607 (24.0)	0.45
Atrial flutter or fibrillation	56 (1.6)	26 (2.7)	30 (1.2)	**<0.01**
Mitral and/or aortic valvular disease	55 (1.6)	19 (1.9)	36 (1.4)	0.27
Diabetes mellitus	342 (9.8)	96 (9.8)	246 (9.7)	0.94
Hypertension	1467 (41.8)	450 (46.0)	1017 (40.2)	**<0.01**
Dyslipidemia	1838 (52.4)	529 (54.1)	1309 (51.8)	0.22
Hyperthyroidism	34 (1.0)	8 (0.8)	26 (1.0)	0.57
Hypothyroidism	633 (18.0)	186 (19.0)	447 (17.7)	0.35
Cirrhosis	4 (0.1)	1 (0.1)	3 (0.1)	0.9
Chronic lung disease	572 (16.3)	191 (19.5)	381 (15.1)	**<0.01**
Diagnosed dementia	5 (0.1)	2 (0.2)	3 (0.1)	0.55
Hospitalized bleed	12 (0.3)	7 (0.7)	5 (0.2)	**<0.05**
**Medications**				
Angiotensin-converting enzyme inhibitor	511 (14.6)	162 (16.6)	349 (13.8)	**<0.05**
Angiotensin II receptor blocker	299 (8.5)	97 (9.9)	202 (8.0)	0.07
Beta blocker	462 (13.2)	140 (14.3)	322 (12.7)	0.21
Calcium channel blocker	283 (8.1)	97 (9.9)	186 (7.4)	**<0.05**
Loop Diuretic	36 (1.0)	16 (1.6)	20 (0.8)	**<0.05**
Thiazide Diuretic	806 (23.0)	250 (25.6)	556 (22.0)	**<0.05**
Alpha ARA	28 (0.8)	10 (1.0)	18 (0.7)	0.35
Any anti-hypertensive agent	1411 (40.2)	437 (44.7)	974 (38.5)	**<0.001**
K-sparing diuretic	12 (0.3)	1 (0.1)	11 (0.4)	0.13
Nitrates	4 (0.1)	1 (0.1)	3 (0.1)	0.9
Vasodilators	9 (0.3)	1 (0.1)	8 (0.3)	0.26
Antiarrhythmic drug	5 (0.1)	3 (0.3)	2 (0.1)	0.11
Statin	1044 (29.8)	308 (31.5)	736 (29.1)	0.17
Other lipid-lowering agent	48 (1.4)	11 (1.1)	37 (1.5)	0.44
Non-aspirin antiplatelet agent	2 (0.1)	0 (0.0)	2 (0.1)	0.38
Non-steroidal anti-inflammatory drug (NSAID)	364 (10.4)	107 (10.9)	257 (10.2)	0.5
Diabetic therapy	124 (3.5)	39 (4.0)	85 (3.4)	0.37
**Vital signs and laboratory values**				
Systolic blood pressure, mmHg				
Mean (SD)	123.0 (15.4)	124.1 (15.5)	122.6 (15.4)	**<0.05**
Median (IQR)	122.0 (112.5–133.0)	123.0 (113.0–134.5)	122.0 (112.0–132.5)	<0.05
Range	63.0–195.5	82.0–188.5	63.0–195.5	
Body mass index, kg/m^2^				
Mean (SD)	27.5 (5.9)	27.6 (5.8)	27.4 (5.9)	0.41
Median (IQR)	26.4 (23.3–30.6)	26.5 (23.4–30.8)	26.4 (23.2–30.6)	0.31
Range	15.8–61.6	15.8–56.7	17.2–61.6	
Waist circumference, cm				
Mean (SD)	86.4 (15.3)	86.1 (14.5)	86.5 (15.5)	0.44
Median (IQR)	84.5 (75.8–95.0)	84.3 (75.9–94.9)	84.6 (75.7–95.2)	0.72
Range	31.3–239.2	31.5–197.5	31.3–239.2	
Overall Ankle-Brachial Index				
Mean (SD)	1.0 (0.1)	1.0 (0.1)	1.0 (0.1)	**<0.001**
Median (IQR)	1.1 (1.0–1.1)	1.0 (1.0–1.1)	1.1 (1.0–1.1)	**<0.001**
Range	0.2–1.6	0.6–1.4	0.2–1.6	
Breast arterial calcification, mg				
Mean (SD)	3.0 (12.8)	10.6 (22.5)	0.0 (0.0)	**<0.001**
Median (IQR)	0.0 (0.0–0.3)	2.7 (0.9–10.7)	0.0 (0.0–0.0)	<0.001
Range	0.0–341.7	0.0–341.7	0.0–0.0	
Estimated GFR, mL/min/1.73 m^2^				
Mean (SD)	80.1 (13.2)	79.0 (13.3)	80.5 (13.1)	**<0.01**
Median (IQR)	81.2 (70.8–90.5)	80.1 (69.4–89.8)	81.5 (71.6–90.9)	<0.01
Range	20.9–118.6	34.6–118.6	20.9–115.8	
Estimated GFR category, mL/min/1.73 m^2^				**<0.001**
90–150	945 (27.0)	235 (24.1)	710 (28.1)	
60–89	2300 (65.6)	648 (66.2)	1653 (65.3)	
45–59	234 (6.7)	90 (9.2)	144 (5.7)	
30–44	26 (0.7)	5 (0.5)	21 (0.8)	
15–29	2 (0.1)	0 (0.0)	2 (0.1)	
Urine albumin-to-creatinine ratio, mg/mmol				
Mean (SD)	9.7 (20.5)	9.9 (18.5)	9.6 (21.3)	0.72
Median (IQR)	5.5 (4.0–8.5)	5.5 (4.1–8.8)	5.5 (4.0–8.3)	0.21
Range	0.7–364.5	1.1–263.3	0.7–364.5	
Calcium, mg/dL				
Mean (SD)	9.8 (0.4)	9.8 (0.4)	9.8 (0.4)	0.89
Median (IQR)	9.7 (9.5–10.0)	9.7 (9.5–10.0)	9.7 (9.5–10.0)	0.84
Range	8.2–14.0	8.6–11.6	8.2–14.0	
Hs-CRP, mg/dL				
Mean (SD)	3.4 (5.8)	3.9 (7.0)	3.2 (5.3)	**<0.01**
Median (IQR)	1.5 (0.7–3.8)	1.6 (0.7–4.1)	1.5 (0.6–3.6)	**<0.05**
Range	0.2–78.8	0.2–76.1	0.2–78.8	
HbA1C, %				
Mean (SD)	5.9 (0.6)	5.9 (0.7)	5.9 (0.6)	0.25
Median (IQR)	5.8 (5.6–6.0)	5.8 (5.6–6.0)	5.8 (5.6–6.0)	0.58
Range	4.5–14.3	4.5–14.3	4.5–13.4	
Hemoglobin, g/L				
Mean (SD)	13.4 (1.0)	13.4 (1.1)	13.4 (1.0)	0.91
Median (IQR)	13.5 (12.8–14.1)	13.5 (12.8–14.1)	13.5 (12.8–14.1)	0.97
Range	7.0–18.6	8.7–17.8	7.0–18.6	
LDL cholesterol, mg/dL				
Mean (SD)	122.0 (32.3)	122.3 (32.8)	121.9 (32.1)	0.74
Median (IQR)	120.0 (100.0–141.0)	120.0 (100.0–141.0)	119.0 (100.0–141.0)	0.84
Range	24.0–332.0	37.0–274.0	24.0–332.0	
Phosphorous, mg/dL				
Mean (SD)	3.6 (0.5)	3.6 (0.5)	3.6 (0.5)	0.55
Median (IQR)	3.6 (3.3–3.9)	3.6 (3.3–3.9)	3.6 (3.3–3.9)	0.52
Range	1.6–5.5	2.2–5.2	1.6–5.5	
PTH, pg/mL				
Mean (SD)	39.2 (23.1)	40.0 (27.8)	38.9 (21.0)	0.2
Median (IQR)	35.0 (25.0–49.0)	35.0 (24.0–49.0)	35.0 (25.0–49.0)	0.84
Range	3.0–425.0	3.0–425.0	3.0–192.0	
Vitamin D, ng/mL				
Mean (SD)	36.1 (12.9)	36.9 (12.7)	35.8 (13.0)	**<0.05**
Median (IQR)	35.0 (28.0–44.0)	36.0 (28.0–45.0)	35.0 (28.0–43.0)	**<0.01**
Range	5.0–227.0	5.0–111.0	5.0–227.0	

### Prevalence of CKD and proteinuria stratified by BAC

**Fig 1:**
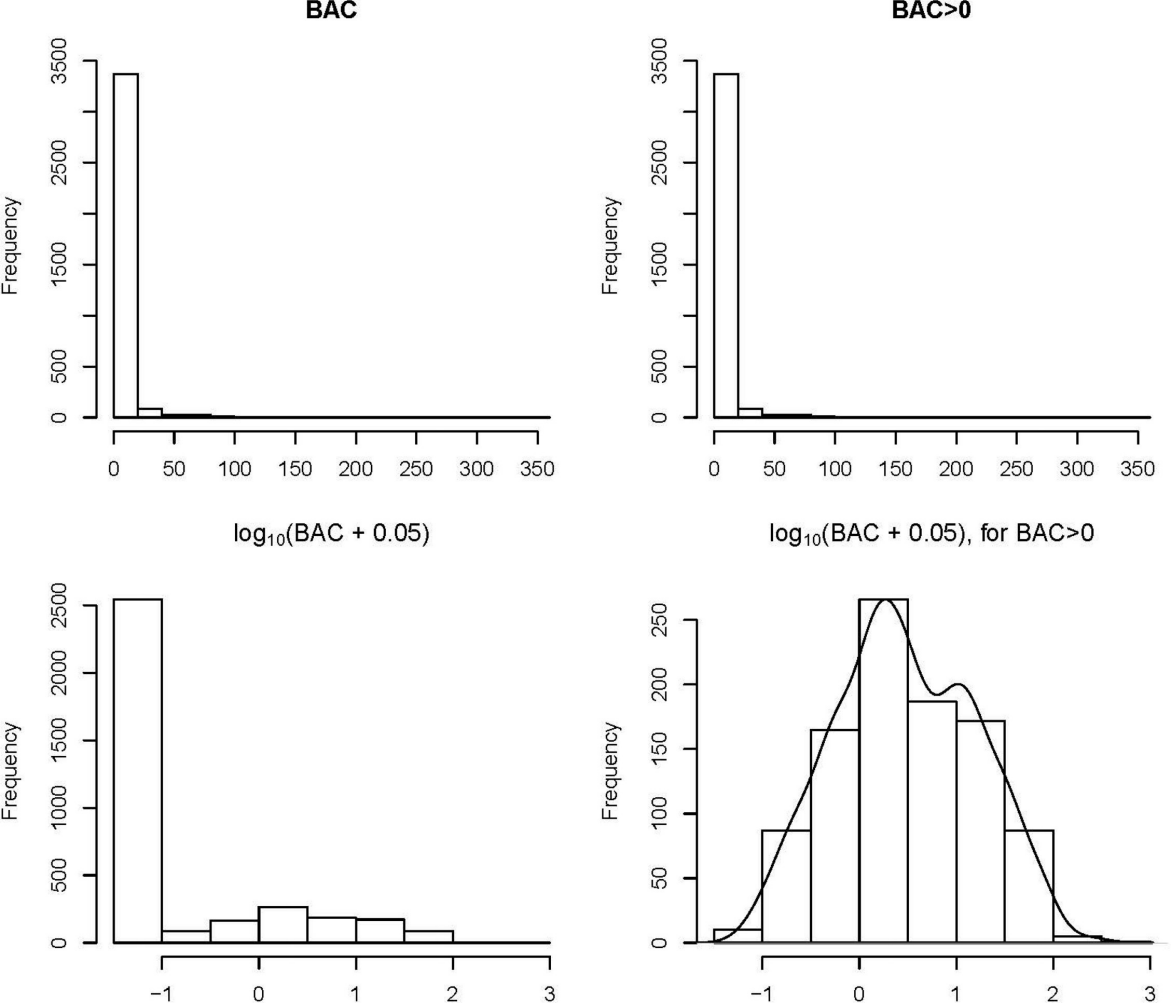
Histograms of BAC (mg) and transformations.

**Fig 2:**
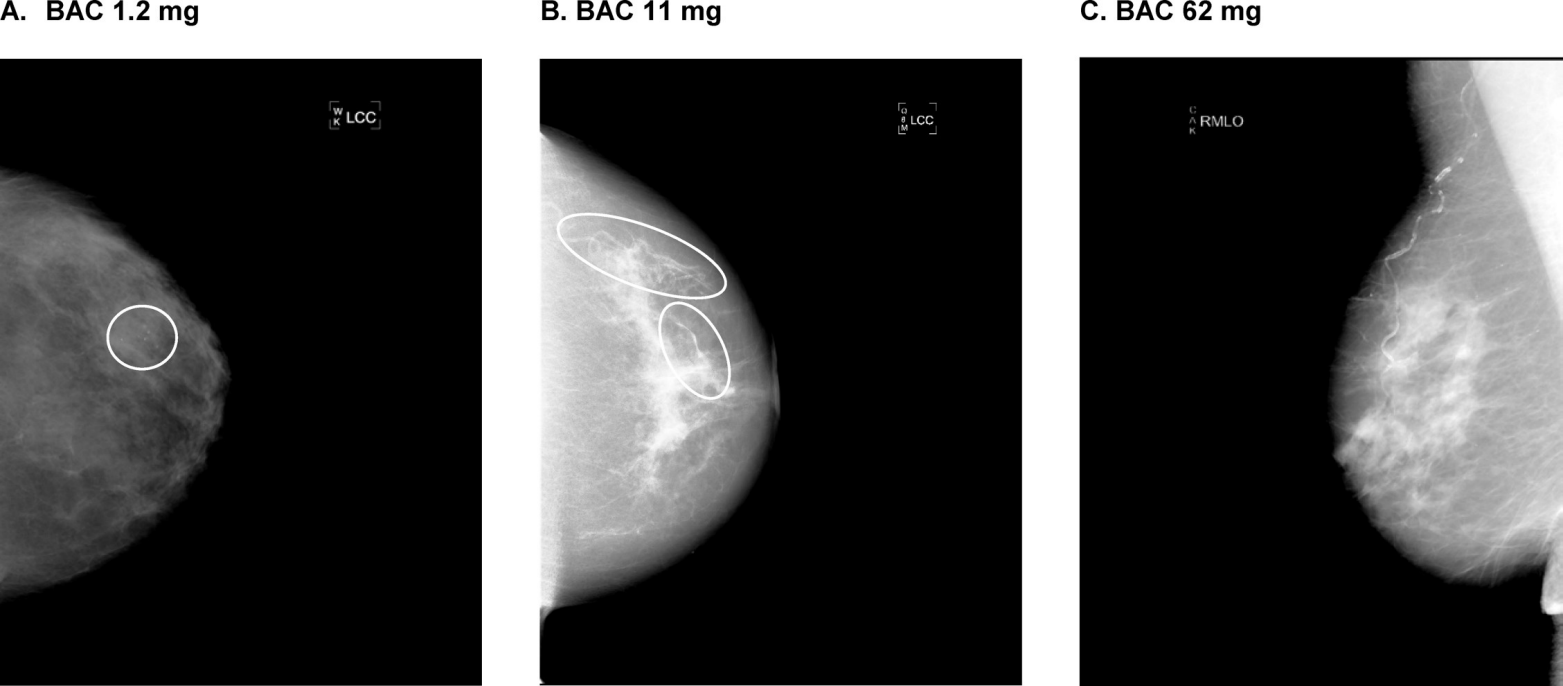
Mammograms for women with minimal BAC (1.2 mg) mild BAC (11 mg) and severe BAC (62 mg).

At entry, 7.5% of participants had CKD, defined as an eGFR of <60 mL/min/1.73 m^2^, with a high prevalence of uACR ≥30 mg/g (85.5%) and a low prevalence of uACR ≥300 mg/g (3.3%). The prevalence of any measured BAC (>0 mg) was 27.9% and 7.5% had BAC >10 mg, with the distribution of BAC mass highly right-skewed among the overall population as well as among the subset with non-zero BAC mass (**Fig 1**). Examples of minimal, mild, and severe BAC can be seen in **Fig 2**.

Among uACR categories, BAC prevalence using either cutoff was not significantly different. However, patients with CKD had significantly higher unadjusted prevalence of BAC at both the 0 mg (*p* = 0.002) and 10 mg (*p* = 0.01) thresholds (**Table 2**).

**Table 2:** Proportions of patients with breast arterial calcification mass stratified by kidney function.

Variable	Overall N (%)	BAC > 0 mg N (%)	*p*-value	BAC ≥ 10 mg N (%)	*p*-value
uACR category, mg/g			0.23		0.82
<30	510 (14.5)	131 (25.7)		37 (7.3)	
≥30	2997 (85.5)	847 (28.3)		226 (7.5)	
uACR category, mg/g			0.36		0.66
<300	3390 (96.7)	941 (27.8)		253 (7.5)	
≥300	117 (3.3)	37 (31.6)		10 (8.6)	
eGFR category, mL/min/1.73m^2^			**0.002**		**0.01**
≥60	3245 (92.5)	883 (27.2)		233 (7.2)	
<60	262 (7.5)	95 (36.3)		30 (11.5)	
Overall	3507	978 (27.9)		263 (7.5)	

#### Multivariable associations of kidney function and proteinuria with BAC

We next conducted logistic regression models for to examine the association of uACR and eGFR with BAC using both thresholds. We found that there was no significant univariate association of uACR ≥30 mg/g with BAC, and results did not materially change after serial adjustment for several categories of potential confounders or mediators (**Table 3**).

**Table 3:** Association of albuminuria (≥30 mg/g) with breast arterial calcification in 3507 women aged 60 to 79 years enrolled in the MINERVA study.

	Odds Ratio (95% Confidence Interval)	
Model	BAC > 0 mg (N = 978)	BAC ≥ 10 mg (N = 263)
uACR only	1.17 (0.94–1.45)	1.09 (0.76–1.56)
+ Sociodemographic characteristics	1.04 (0.83–1.30)	0.96 (0.66–1.40)
+ Reproductive history	1.03 (0.82–1.29)	0.93 (0.63–1.36)
+ Lifestyle factors	1.03 (0.82–1.29)	0.92 (0.63–1.36)
+ Cardiovascular risk factors and disease	1.02 (0.81–1.28)	0.87 (0.59–1.29)
+ Other medical history	1.03 (0.82–1.29)	0.89 (0.60–1.32)
+ Medications	1.02 (0.81–1.28)	0.89 (0.60–1.32)
+ Potential mediating variables	1.02 (0.81–1.28)	0.88 (0.59–1.31)

Sociodemographic: age, race, educational attainment, household income, marital status, and whether the patient was born in the US. Lifestyle factors: use of alcohol in the past year, smoking status, and hours of physical activity per week. Reproductive history: age at menarche, age periods stopped, polycystic ovary syndrome, receipt of menopausal hormone therapy, history of breastfeeding, and number of children. Cardiovascular risk factors and diseases: atrial fibrillation or flutter, mitral and/or aortic valvular disease, diabetes mellitus, hypertension, and dyslipidemia, ankle-brachial index. Medical history: prior migraine, gout, osteoporosis, osteoarthritis, depression, hyperthyroidism, hypothyroidism, cirrhosis, chronic lung disease, hospitalized bleeds. self-reported health status, and parental history of stroke, heart failure, heart attack. Medications: angiotensin converting enzyme (ACE) inhibitors, angiotensin II receptor blockers (ARBs), β-blockers, calcium channel blockers, diuretics, aldosterone receptor antagonists, alpha blockers, antiarrhythmic agents, nitrates, other vasodilators, non-aspirin antiplatelet agents, statins, other lipid-lowering agents, anti-diabetic agents, and non-steroidal anti-inflammatory drugs. Mediators: systolic blood pressure, body mass index, waist circumference, and calcium, hs-CRP, HBA1c, hemoglobin, LDL cholesterol, phosphorous, parathyroid hormone, and vitamin D levels.

In unadjusted models, we found that CKD was associated with higher odds of BAC >0 mg (OR: 1.53, 95% CI:1.18–2.00) and BAC >10 mg (OR: 1.66, 95% CI:1.11–2.49) (**Table 4**). However, these associations were attenuated and no longer statistically significant with the addition of sociodemographic characteristics (**Table 4**). Additional adjustment for other potential confounders or mediators did not materially affect the observed associations. We observed a stronger unadjusted association between CKD and BAC >0 mg among 412 black participants (OR: 2.80, 95% CI:1.32–5.95), but this association was attenuated and no longer significant after adjustment for sociodemographic characteristics. There was no significant crude association between CKD and BAC in Hispanic or Asian/Pacific Islander participants. In sensitivity analyses using linear regression analyses between continuous eGFR or log-transformed uACR with log-transformed BAC, we also found no significant associations. In the case-control analysis, the 262 CKD patients were successfully matched to 262 non-CKD patients on age and race categories. Among CKD patients, 95 (36.3%) had BAC >0 mg and 30 (11.5%) had BAC >10 mg. Among non-CKD patients, 82 (31.3%) had BAC >0 mg and 24 (9.2%) had BAC >10 mg. The odds ratio for BAC >0 mg was 1.25 (95% CI: 0.85–1.83) and for BAC >10 mg was 1.28 (95% CI: 0.70–2.37), both indicating a non-statistically significant difference in BAC between CKD patients and non-CKD patients.

**Table 4:** Association of reduced estimated GFR (<60 mL/min/1.73m^2^) with breast arterial calcification in 3507 women aged 60 to 79 years enrolled in the MINERVA study.

	Odds Ratio (95% Confidence Interval)	
Model	BAC > 0 mg (N = 978)	BAC ≥ 10 mg (N = 263)
eGFR only	1.53 (1.18–2.00)	1.66 (1.11–2.49)
+ Sociodemographic characteristics	1.28 (0.97–1.69)	1.30 (0.84–1.99)
+ Reproductive history	1.26 (0.95–1.66)	1.20 (0.77–1.88)
+ Lifestyle factors	1.24 (0.94–1.65)	1.13 (0.72–1.77)
+ Cardiovascular risk factors and disease	1.23 (0.93–1.64)	1.13 (0.71–1.78)
+ Other medical history	1.25 (0.94–1.67)	1.14 (0.72–1.80)
+ Medications	1.28 (0.96–1.70)	1.22 (0.77–1.92)
+ Potential mediating variables	1.22 (0.91–1.63)	1.15 (0.72–1.84)

Sociodemographic: age, race, educational attainment, household income, marital status, and whether the patient was born in the US. Lifestyle factors: use of alcohol in the past year, smoking status, and hours of physical activity per week. Reproductive history: age at menarche, age periods stopped, polycystic ovary syndrome, receipt of menopausal hormone therapy, history of breastfeeding, and number of children. Cardiovascular risk factors and diseases: atrial fibrillation or flutter, mitral and/or aortic valvular disease, diabetes mellitus, hypertension, and dyslipidemia, ankle-brachial index. Medical history: prior migraine, gout, osteoporosis, osteoarthritis, depression, hyperthyroidism, hypothyroidism, cirrhosis, chronic lung disease, hospitalized bleeds. self-reported health status, and parental history of stroke, heart failure, heart attack. Medications: angiotensin converting enzyme (ACE) inhibitors, angiotensin II receptor blockers (ARBs), β-blockers, calcium channel blockers, diuretics, aldosterone receptor antagonists, alpha blockers, antiarrhythmic agents, nitrates, other vasodilators, non-aspirin antiplatelet agents, statins, other lipid-lowering agents, anti-diabetic agents, and non-steroidal anti-inflammatory drugs. Mediators: systolic blood pressure, body mass index, waist circumference, and calcium, hs-CRP, HBA1c, hemoglobin, LDL cholesterol, phosphorous, parathyroid hormone, and vitamin D levels.

## Discussion

Within a contemporary, ethnically diverse cohort of 3,507 women with no prior clinical CVD who were undergoing regular screening mammography, we found that quantifiable BAC (>0 mg) was present in 28% of participants which was higher than reported in previous studies, and approximately 1 in 12 participants had BAC >10 mg.[14] In addition to differences in our population compared with previously studied samples, an important feature in our study was the use of digital quantification of mammograms which has a higher sensitivity of BAC detection compared with visual inspection.[26] We observed no significant association between albuminuria with two different BAC thresholds in univariate or multivariable analyses. While there was a higher crude association of CKD with BAC >0 mg or BAC >10 mg, the relationship was attenuated and no longer statistically significant after adjustment for sociodemographic factors. Among blacks, the unadjusted association between CKD and BAC was stronger than in the overall population, but the association was no longer significant after accounting for differences in other sociodemographic characteristics. Furthermore, findings remained consistent in a variety of sensitivity analyses evaluating exposures of kidney function and albuminuria as well as BAC as continuous log-transformed measures.

To date, few studies have examined the association of eGFR or albuminuria with BAC. Hassan et al. studied 292 patients with CKD or ESRD from 2006–2011, along with the same number of controls matched on age, race and diabetes status.[19] Presence or absence of BAC was assessed through visual inspection of mammograms, and the prevalence of any BAC was significantly higher in those with Stage 4 CKD or higher. Estimated GFR was a significant predictor of BAC; in multivariable analyses accounting for age, race, and diabetes status, there was an association between lower eGFR and any BAC (adjusted odds ratio per ml/min/1.73m^2^ increase 0.96, 95%CI:0.92–0.99).[19] However, this study was limited by a small sample size and limited ability to adjust for potential confounders. Other studies of BAC involving populations with reduced kidney function largely focus on ESRD patients receiving dialysis, or provide only descriptive results without multivariable adjustment. Duhn et al. found that the prevalence of any BAC (based on visual inspection of mammograms) was 36% in their sample of patients with CKD or ESRD, but only 14% in patients with stage 3 CKD, further suggesting that BAC may only be associated with more advanced stages of CKD and ESRD.[13] A more recent study by Manzoor et al. investigated the progression of BAC in 137 CKD patients with eGFR <90 mL/min/1.73m^2^ and found that progression of BAC was not increased in patients with an eGFR ≥40 mL/min/1.73m^2^.[40] This is consistent with our findings, given that our CKD population consisted of primarily lower-severity patients. To our knowledge, our study is the first study exploring the potential relationship between proteinuria and BAC using standardized measures of uACR and BAC.

Previous studies of kidney dysfunction suggest a positive association with intimal calcification of the coronary arteries and valves,[3–7, 10] but the relation with medial calcification and calcification of other non-coronary vascular beds has been less well studied. Other studies also suggest that BAC may not be consistently associated with several common CVD risk factors (e.g., hypertension, obesity, dyslipidemia, and smoking).^14–18^ Several potential mechanisms have been identified in which CKD may influence vascular calcification, including dysregulation of mineral bone metabolism, with recent studies suggesting osteogenic transdifferentiation in vascular smooth muscle cells leading to calcification, although this may not affect all vascular beds in the same manner.[5, 11, 20]

While our findings support that kidney function and proteinuria may have differential associations with different arterial distributions, we believe that our findings should be considered in the context of several limitations. First, despite enrolling a diverse and well-characterized cohort, by design, we excluded patients with any known clinical CVD which likely affected the prevalence and severity of BAC as well as the representation of more severe CKD or albuminuria. In our cohort, we observed a relatively low presence of CKD (7.5%) with the majority of CKD patients having an eGFR between 45–59 mL/min/1.73m^2^ (89.2% of CKD patients). This may reduce the generalizability of our results to more advanced CKD populations. Our participants were derived from an integrated health care delivery system in northern California that is heavily focused on primary prevention as reflected by the relatively high use of antihypertensive and lipid-modifying therapies shown in Table 1. Collectively, this may have affected our ability to detect meaningful associations between kidney dysfunction and BAC. Because our quantitative measure of BAC was a novel, continuous measure, the clinically relevant threshold of BAC using this approach has not been definitively determined. Finally, our results may not be fully generalizable to the uninsured, other geographic areas or all practice settings.

In sum, among women with no known CVD undergoing regular mammography screening, we found that reduced eGFR and albuminuria were not significantly associated with BAC. Future studies should evaluate whether BAC influences the risk of future CVD events across the spectrum of kidney function.
